# Molecular and Cellular Pathways as a Target of Therapeutic Hypothermia: Pharmacological Aspect 

**DOI:** 10.2174/157015912799362751

**Published:** 2012-03

**Authors:** Hyung Soo Han, Jaechan Park, Jong-Heon Kim, Kyoungho Suk

**Affiliations:** 1 Department of Physiology, Brain Science & Engineering Institute, Kyungpook National University School of Medicine, Daegu, 700-422, Korea; 2 Department of Neurosurgery, Brain Science & Engineering Institute, Kyungpook National University School of Medicine, Daegu, 700-422, Korea; 3 Department of Pharmacology, Brain Science & Engineering Institute, Kyungpook National University School of Medicine, Daegu, 700-422, Korea

**Keywords:** Hypothermia, pharmacotherapy, drug target, signal pathway, neuroinflammation.

## Abstract

Induced therapeutic hypothermia is the one of the most effective tools against brain injury and inflammation. Even though its beneficial effects are well known, there are a lot of pitfalls to overcome, since the potential adverse effects of systemic hypothermia are still troublesome. Without the knowledge of the precise mechanisms of hypothermia, it will be difficult to tackle the application of hypothermia in clinical fields. Better understanding of the characteristics and modes of hypothermic actions may further extend the usage of hypothermia by developing novel drugs based on the hypothermic mechanisms or by combining hypothermia with other therapeutic modalities such as neuroprotective drugs. In this review, we describe the potential therapeutic targets for the development of new drugs, with a focus on signal pathways, gene expression, and structural changes of cells. Theapeutic hypothermia has been shown to attenuate neuroinflammation by reducing the production of reactive oxygen species and proinflammatory mediators in the central nervous system. Along with the mechanism-based drug targets, applications of therapeutic hypothermia in combination with drug treatment will also be discussed in this review.

## 1 PHARMACOLOGICAL TARGETS FOR DRUG DEVELOPMENT 

Alteration of body temperature can disturb the homeostasis of our body system, and hypothermia is known to make changes at the molecular , cellular, and system levels. At the cellular and system levels, hypothermia has been reported to protect neurons from injury, prevent activation of glial cells (especially microglia), and attenuate endothelial and blood-brain barrier (BBB) dysfunction. Since microglia are the major cells mediating inflammation and BBB maintains the neurovascular integrity in brain, protective mechanisms of therapeutic hypothermia range from direct protection of neurons against primary insults to prevention of secondary injury mediated by inflammation and blood brain barrier abnormalities. At the molecular level, most hypothermic effects can be explained by alterations in the cellular signaling pathways, gene regulating systems, and microstructures of cells. Some of these molecular events are directly triggered by hypothermia, while the majority of alterations are secondary or indirect consequences of hypothermia. When therapeutic hypothermia is applied to brain injury models such as traumatic brain injury, stroke, cardiac arrest, perinatal asphyxia, aortic aneurysm surgery, etc, it becomes more complex due to an abnormal pathological environment in the damaged brain tissue. Therefore, we have to take the interference of injury-related effects into consideration in addition to the hypothermia itself. Even though systematic investigation has not been done yet, there is possibility that the hypothermic mechanisms or effects may be different depending on the depth of cooling as well. In general, hypothermia is classified as mild (32-35°C), moderate (28-32°C) and severe (less than 28°C), and most of the hypothermia studies were done in the range of mild to moderate hypothermia. Elucidation of the protective mechanisms of hypothermia may provide precious clues that may help develop protective drugs in the future. Nevertheless, ample evidence indicates that suppression of deleterious neuroinflammation is one of the major protective mechanisms of therapeutic hypothermia in brain.

### 1.1 Signal Pathways

It is well established that hypothermia induces changes in many intracellular signaling cascades. In general, hypothermia has suppressive effects, but some signal systems are activated by hypothermia. Most commonly reported signal systems might be mitogen activated protein kinase (MAPK) pathway. MAPKs are serine/threonine-specific protein kinases that respond to extracellular stimuli such as mitogens, stress or shock, inflammatory stimuli, etc. MAPKs regulate various cellular activities including gene induction, cellular survival and death, etc. Depending on the experimental conditions, hypothermia exerts different effects on MAPKs. In some cases, hypothermia is known to activate extracellular signal-regulated kinases-1/2 (ERK1/2) and promotes protection [1-6]. In other cases, hypothermia has been reported to block the activation of ERK1/2 and suppresses inflammation and cell death [7]. In a recent review by Sawe and colleagues (2008), the dual role of ERK1/2 after the occurrence of a stroke has been described [8]. Taken these data together, hypothermia seems to potentiate protective effects of ERK1/2 and diminish deleterious effects of ERK1/2. Regarding Jun N-terminal kinase (JNK), another member of the MAPK family, it has been reported that hypothermia protects ischemic endothelial cells [9,10], traumatic brain injury [11] and astrocytes against injury [12] *via *the early suppression of JNK activation and the subsequent prevention of apoptosis or cell death. The effect of hypothermia on p38 is similar to that on JNK. By inhibiting p38 activation, hypothermia suppresses inflammation [13], apoptosis and endothelial dysfunction [14]. These studies suggest that inhibition of p38 or components of the p38 pathways could be a promising and selective anti-inflammatory therapeutic target of hypothermic application. In summary, mild hypothermic effects are mediated by the suppression of deleterious components of the MAPK pathways and the promotion of protective MAPK signals. In cases of severe hypothermia, however, hypothermic stress in endothelial cells is mediated by p38, ERK1/2 and nuclear factor kappa B (NFκB) [15]. Therefore, the idea of protective signals should be limited to mild therapeutic hypothermia, but not severe hypothermia.

NFκB is a protein complex that controls the transcription of DNA. NFκB is found in almost all animal cell types and is involved in cellular responses to a variety of stimuli. NFκB plays a key role in regulating the immune response and inflammation. In many reports, hypothermia downregulates inflammation-related NFκB actions [16-21]. However, some studies showed hypothermic augmentation of NFκB activation [22-25]. These contrasting effects are not clearly understood yet. Since NFκB has both proinflammatory and protective effects at the same time, hypothermia may interfere with different aspects of NFκB pathways depending on the cell types or experimental conditions. Recently, enhanced expression of NFκB during the recovery from deep hypothermia was reported [26-28]. Therefore, the cellular mechanisms underlying hypothermia-induced NFκB actions including the effects during the rewarming process need to be further investigated. 

Signal transducer and activator of transcription (STAT) is a family of transcription factors involved in many cellular activities. In ischemic models, STAT3 and STAT5 are known to mediate neuroprotection against ischemia [29, 30]. STATs are also involved in ischemic preconditioning-induced mitochondria mediated-neuroprotection [31]. In microglia, STAT signaling mediates inflammation [32]. Regarding the hypothermic effect, it is reported that normothermic ischemia-reperfusion caused a significant increase in phosphorylated STAT-1 and STAT-3 when compared with normothermic sham; both of these increases were completely abolished by moderate hypothermia [33]. Choi *et al*. also reported hypothermic attenuation of STAT-3 phosphorylation and intercellular adhesion molecule-1 induction in the vascular system of stroke models [3]. Contrasting data was reported in severe hypothermic conditions [34]. They demonstrated that the anti-inflammatory reactions observed in hypothermia during cardiopulmonary bypass were associated with up-regulated interleukin-10 *via *STAT-3 activation, which in turn led to the attenuation of tumor necrosis factor-alpha expression and to hepatic protection. In these cases, even though the anti-inflammatory effect of hypothermia was the same, the underlying mechanisms were different depending on the organ and hypothermic depth. 

Also, studies on survival signals such as Akt and phosphatase and tensin homolog deleted on chromosome 10 (PTEN) have been reported. Akt is a serine/threonine protein kinase that plays a key role in multiple cellular processes such as glucose metabolism, cell proliferation, apoptosis, transcription and cell migration. The effect of hypothermia on survival signal Akt was not significant in the neonatal hypoxic brain but slightly depressed [35], while activation of Akt contributed to the protection of cardiac muscles [36]. PTEN, one of the well-known survival factors, has been associated with hypothermic protection [37]. In addition to the promotion of survival signals, protection can be achieved by preventing death signals. Hypothermic inhibition of the increased assembly of the GluR6-PSD95-MLK3 signaling module induced by cerebral ischemia/reperfusion is one such type of protection mechanism [38]. Hypothermia also reduced oxidative DNA damage and pro-death signaling events in rat models of cerebral ischemia [39]. Other signal pathways such as calcium and adenosine triphosphate have also been shown to be altered by hypothermia. Mild hypothermia delayed neuronal damage by enhancing the restoration of the Ca2+/calmodulin-dependent protein kinase II-mediated cell signaling [40]. Severe hypothermia significantly increased phosphorylation state of AMP-dependent activated protein kinase [41]. Hypothermia seems to give rise to contrasting outcomes, partly because the experiments were performed in different hypothermic conditions. Discrepancies in the duration and depth of hypothermia might lead to different effects on signal systems. Differences of the injury models might also contribute to the confusing results as well. 

### 1.2 Gene Expression 

The protective mechanisms of therapeutic hypothermia have been investigated using gene expression analysis tools. Recently, microarray studies provided an extensive amount of data at the transcriptome levels [42-47]. These studies provided large-scale data but a lack of sophisticated analysis tools hindered the systemic integration of the massive microarray data. So far, it does not seem to be easy to extract well-organized and user-friendly diagrams of neuroprotection mechanisms from the microarray study. However, they suggested lots of candidate genes and it will take time to investigate the functional implications of the candidate genes. Along with the mass screening, traditional studies on the specific target genes have been steadily undertaken. These studies usually focus on genes related with well-known pathways such as cell death or survival [4, 39, 48-50], inflammation [51-57], and free radicals [58-60]. Although inflammation may be a sequel of brain injury as well as infection, injury-related inflammation is a focus of the current reivew. Most of the studies have consistently demonstrated that hypothermia prevents or reduces deleterious pathways while enhancing survival or protective events. However, there is an exception to this norm. In contrast to the general idea that lowered temperature might decrease reactive oxygen species (ROS) production, it has been reported that hypothermia decreased the rates of resting and phosphorylating respiration phases and this respiratory slowdown was associated with an increase in ROS production, hydrogen peroxide release and carbon-centered lipid radicals in isolated mitochondria at 32°C and 35°C [61]. We don’t have a clear explanation for this contrasting data but it is evident that analyses of mechanisms at the molecular level cannot predict the clinical outcome of the patients. That being said, mechanism-based drug development should focus on these target genes. 

The transcription factor is a protein that binds to specific DNA sequences, thereby controlling the transcription of genetic information from DNA to mRNA. Transcription factors perform their role in a complex with other proteins including RNA polymerase. They are important regulators of gene expression and thereby control cellular events by making functional proteins or microRNAs. Early studies are focused on immediate early genes such as c-fos, c-jun, zif/268, pCREB and AP-1 [62-66]. Hypoxia-inducible factors (HIFs) are transcription factors that respond to changes in available oxygen in the cellular environment, specifically, to hypoxia. Hypothermia is known to suppress HIF-1 alpha protein synthesis and HIF-1 mediated gene expression [67]. Considering the relatively broad and nonspecific actions, it will not be easy to develop neuroprotectants based on transcription factors. Some of the transcription factors, such as NFκB and STATs, were also mentioned in the “signal pathways” section, as signal systems are closely related with transcription factors.

### 1.3 Cellular Structure

By being exposed to hypothermia, cells undergo death or adaptation process depending on the degree of lowered temperature [68]. Lipids are the major components of the cell and change their functions and structures in response to physical conditions. The lipid composition of the cell membrane changes with alterations in the environment, and this change depends on the synthesis, metabolism and intermembrane transfer of the lipid. It was shown that lipids of membranes and cellular organelles of mammalian brain are involved in the adaptation to low temperatures [69]. In normal rat brains, the nuclear fractions of neurons and neuroglia are depleted of phospholipids and cholesterol and enriched in mono- and diglycerides and fatty acids. In the hypothermic state, the proportion of phosphatidylinositol in the microsomal fraction decreases. In the nuclei of glial cells, the content of cholesterol and the ratio between cholesterol and phospholipids increases [70]. It has also been reported that hypothermia increases lipolysis, free fatty acid turnover rate and triglyceride/free fatty acid cycling in the human body so as to amplify the ability of stored triglycerides to react quickly to major changes in energy expenditure induced by a sustained cold environments [71]. 

Along with the lipid changes, other cellular structures are also affected by hypothermia. In neurons, several key aspects of synaptic transmission, including the distribution of postsynaptic neurotransmitter receptors, postsynaptic ion fluxes, and the spread of presynaptically released neurotransmitters, are strongly influenced by the shapes of dendritic spines. Hypothermia is known to induce changes in the shape of dendritic spines [72]. Dendritic spines are highly sensitive to hypothermia and rapidly lose actin-based motility followed by the reversible loss of the entire spine structure. Thus, reduced temperature significantly affects synaptic morphology, which is in turn known to influence synaptic transmission. Hypothermia-induced spine morphology changes may have a significant influence on brain function [73, 74]. The changes of lipid composition and cellular structures are mostly observed in severe hypothermic conditions. However, sparing effects of mild hypothermia on dendritic spine morphology after *in vitro* ischemia was also reported, and the underlying mechanism was found to be related with actin redistribution [75]. 

When over-expressed in neuronal cell lines, RNA-binding motif protein 3 (RBM3) enhanced global translation, the formation of active polysomes, and the activation of initiation factors [76]. Since RBM3 is one of the cold inducible mRNA binding proteins, these data suggest that RBM3 plays a distinctive role in enhancing translation in neurons under hypothermic conditions. In addition to protein translation, post-translational modifications of proteins by hypothermia have been also reported [77-80] and some of these protein modifications were related to the neuroprotective mechanism. Hypothermic effects were demonstrated in protein interaction, enzyme activity and the translocation of proteins as well [81, 82]. Some of these hypothermic effects were related to neuroprotection [83], oligodendrocyte precursor cell increase [84], and neurite outgrowth [85]. Rewarming following hypothermia also induces injury related changes [86] and should be taken into consideration in cases of hypothermia applications. 

The blood-brain barrier (BBB) is a very important target of hypothermia in brain injury models. By maintaining the integrity of the BBB, the normal brain environment can be preserved. In case of brain injury, damaged BBB by primary insult cannot prevent the secondary injury by brain edema and increased intracranial pressure is life-threatening. The effect of hypothermia on the BBB has been studied on multiple occasions [87-91]. It has been consistently demonstrated that hypothermia preserves the BBB but the molecular mechanisms of hypothermia on the BBB have not been studied intensively so far. Hypothermia protects the function and structure of BBB from the insult by attenuating the loss of vascular basement proteins [92] and the other proteins constituting BBB [93, 94], reducing matrix metalloproteinase (MMP) [95, 96] and increasing the expression of tissue inhibitors [95]. Aside from the direct protection of BBB integrity, hypothermia affects pericyte migration [97], multi-drug resistance protein 1-mediated transepithelial transport of drugs [98], and transcription factor expression in the BBB [99]. BBB breakdown increases the expression of aquaporin-4 (AQP-4) and mild hypothermia significantly reduces brain edema formation after intracranial hemorrhage by suppressing the elevation of AQP-4 protein expression [100]. These BBB preserving effects of hypothermia can play a pivotal role in novel drug development against many brain injuries.

## 2 COMBINATION OF PHARMACOTHERAPY AND INDUCED HYPOTHERMIA

Patients with traumatic brain injury or stroke often receive extensive drug treatment. When pharmacotherapy is combined with an induced hypothermic treatment, the drug-hypothermia interaction should be considered. As the cooling of the whole body may influence drug metabolism, clearance, and receptor properties, local hypothermia may be the preferable method of brain cooling so as to protect the patients from a variety of central nervous system (CNS) injuries.

### 2.1 Effects of Therapeutic Hypothermia on Pharmacokinetics and Pharmacodynamics

**Fig. (1):**
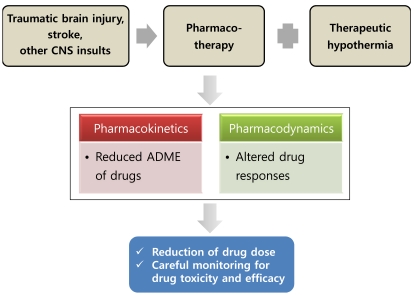
**Effects of therapeutic hypothermia on pharmacokinetic and pharmacodynamic parameters.** Upon traumatic brain injury, stroke, and other brain insults, patients may receive pharmacotherapy as well as hypothermic treatment, which may influence the kinetic as well as dynamic parameters of pharmacotherapy. Patients should be carefully monitored to avoid drug toxicity and treatment failure. ADME; absorption, distribution, metabolism, and elimination.

Critically-ill patients who receive hypothermic treatment are likely to be subject to an extensive phamacotherapeutic regimen as well. A systematic review of preclinical and clinical studies on the effects of hypothermia on pharmacokinetic and pharmacodynamic parameters has recently been published [101]. In their review, the authors stated that therapeutic hypothermia may cause alterations in both the pharmacokinetic and pharmacodynamic aspects of the drugs, which could result in an increased risk of drug toxicity or therapy failure. It can be expected that therapeutic hypothermia influences drug metabolism and clearance as well as drug target location and sensitivity (Fig. **1**). Rewarming to normothermia may also exert similar pharmacological effects. Hence, phamacotherapeutic regimens should be closely monitored to avoid toxicity and therapy failure in patients treated with hypothermia. Tortorici *et al.* recently examined the effects on hypothermia on drug disposition, metabolism, and the response of drugs commonly used in the intensive care unit, with a focus on the cytochrome P450 enzyme system [102]. Mild to moderate hypothermia decreased the systemic clearance of cytochrome P450 metabolized drugs between approximately 7% and 22% per degree Celsius below 37°C during cooling. Hypothermia also decreased the potency and efficacy of certain drugs, thereby narrowing the therapeutic index. As the integrated effect of hypothermia on the pharmacokinetic and pharmacodynamic properties of individual drugs is unclear, further investigation is required to delineate the precise mechanisms of the effect of hypothermia on drug disposition and response, as well as to establish safe and effective drug dosing guidelines during hypothermic treatment [103].

### 2.2 Hypothermia-Drug Interaction: Exogenous or Endogenous Regulation of Therapeutic Hypothermia

Multiple mechanisms of hypothermia-induced neuroprotection have been proposed including its effects on metabolic and hemodynamic consequences, excitotoxicity, BBB, calcium-dependent intracellular signaling, inflammation, edema, and neuronal cell death [104]. These therapeutic effects of hypothermia and the underlying mechanisms can be modulated by a variety of exogenous or endogenous factors. Natural product ginkgolides have been shown to affect the ability of hypothermia to protect astrocytes against hypoxia/reperfusion-induced injury [105]. Schmitt *et al.* demonstrated that the steroid methylprednisolone attenuates hypothermia/rewarming-induced cytotoxicity and IL-6 release in astrocytes, neurons, and microglial cells in culture [106]. Among the endogenous factors, the glial calcium binding protein S100B differentially influenced the cytokine release and cytotoxicity of distinct brain cell types by suppressing hypothermia-induced axonal outgrowth [107]. An anti-inflammatory, cytokine IL-10 suppressed the beneficial effects of moderate hypothermia in rat models of traumatic brain injury [108]. Peripheral benzodiazepine receptor (PBR) and its ligand have also been implicated in therapeutic hypothermia [109]. Mild hypothermia prevented the insult-induced astrocytic expression of PBR and halted the rise of neurosteroid content, thereby reducing astrocyte swelling and edema. Previous *in vitro* studies using cultured astrocytes indicated that PBR and neurosteroids affect ammonia-induced astrocyte swelling.

### 2.3 Drug-Induced Hypothermia: A Focus on the Cannabinoid System

Cannabinoids are a group of chemicals found in plants (phytocannabinoids), the nervous/immune systems of animals (endocannabinoids) or come from synthetic origin (synthetic cannabinoids). Cannabinoids control neuronal excitability at various synapses, participating in the regulation of various physiological processes such as mood, appetite, memory, pain perception, and motor activity [110-112]. These effects of cannabinoids are exerted by their binding to two receptors, cannabinoid receptor type-1 (CB1R) and type-2 (CB2R). CB2R is expressed in microglial cells, astrocytes, and cerebromicrovascular endothelial cells [113], and may modulate the immune and inflammatory responses [114, 115]. CBR1 expression increases with the occurrence of neurodegenerative disease or ischemia [116]. Most importantly, CBR1 has been shown to mediate the hypothermia-inducing effects of cannabinoids [117]. Leker *et al.* demonstrated that the CB1 agonist HU-210 is capable of reducing ischemic damage by inducing hypothermia [118]. CBR1 increases GABA release in the anterior hypothalamic nucleus, the central locus of thermoregulation [117, 119]. The neuroprotective effects of cannabinoids against a variety of insults are believed to be associated with drug-induced hypothermia [120]. Most studies, however, are based on rodent models, raising the question of whether the drug-induced hypothermia is feasible in a large organism. Recently, Fosgerau *et al.* reported that a component of chili pepper, dihydrocapsaicin (DHC), induced mild therapeutic hypothermia in large animals such as monkey and cattle, indicating that drug-induced hypothermia can also be obtained by transient receptor potential vanilloid type 1 agonists in a large animal [121]. 

## 3 CONCLUSIONS AND PERSPECTIVES

Therapeutic hypothermia can be applied to patients either systemically or locally. Because systemic hypothermia may generate side effects such as infection, arrhythmia, hypokalemia, and coagulopathies in some cases, local hypothermia can be considered as an alternative cooling method. Despite wide-spread interest in therapeutic hypothermia, the precise molecular mechanism(s) of its protective effects are largely unknown. Considering the potential adverse effects of systemic hypothermic therapy, the discovery of new therapeutic targets based on mechanistic studies would facilitate the development of new drugs with less side effects and higher efficacy. Furthermore, when considering the potential effects of systemic hypothermia on pharmacokinetics and pharmacodynamics, local hypothermia would deliver a safer and more effective neuroprotection in patients undergoing drug therapy. Hence, a combination of pharmacotherapy and local hypothermia will be the focus of future investigation. One of the major mechanisms underlying therapeutic hypothermia is the attenuation of neuroinflammation. In combination with many anti-inflammatory agents already found [122], local hypothermia may provide a synergistic neuroprotective strategy. Moreover, hypothermia-inducing drugs such as cannabinoids and dihydrocapsaicin may also supplement the localized cooling strategy for optimal neuroprotection.
